# NMDA receptor-mediated CaMKII/ERK activation contributes to renal fibrosis

**DOI:** 10.1186/s12882-020-02050-x

**Published:** 2020-09-09

**Authors:** Jingyi Zhou, Shuaihui Liu, Luying Guo, Rending Wang, Jianghua Chen, Jia Shen

**Affiliations:** 1 Kidney Disease Center, the First Affiliated Hospital, College of Medicine, Zhejiang University, Qingchun Road 79, Hangzhou, 310003 China; 2 Key Laboratory of Nephropathy, Hangzhou, Zhejiang Province China; 3 Kidney Disease Immunology Laboratory, the Third-Grade Laboratory, State Administration of Traditional Chinese Medicine of China, Hangzhou, China; 4 Key Laboratory of Multiple Organ Transplantation, Ministry of Health of China, Hangzhou, China

**Keywords:** CaMKII, ERK, NMDA receptor, Renal fibrosis

## Abstract

**Background:**

This study aimed to understand the mechanistic role of *N*-methyl-D-aspartate receptor (NMDAR) in acute fibrogenesis using models of in vivo ureter obstruction and in vitro TGF-β administration.

**Methods:**

Acute renal fibrosis (RF) was induced in mice by unilateral ureteral obstruction (UUO). Histological changes were observed using Masson’s trichrome staining. The expression levels of NR1, which is the functional subunit of NMDAR, and fibrotic and epithelial-to-mesenchymal transition markers were measured by immunohistochemical and Western blot analysis. HK-2 cells were incubated with TGF-β, and NMDAR antagonist MK-801 and Ca^2+^/calmodulin-dependent protein kinase II (CaMKII) antagonist KN-93 were administered for pathway determination. Chronic RF was introduced by sublethal ischemia–reperfusion injury in mice, and NMDAR inhibitor dextromethorphan hydrobromide (DXM) was administered orally.

**Results:**

The expression of NR1 was upregulated in obstructed kidneys, while NR1 knockdown significantly reduced both interstitial volume expansion and the changes in the expression of α-smooth muscle actin, S100A4, fibronectin, COL1A1, Snail, and E-cadherin in acute RF. TGF-β1 treatment increased the elongation phenotype of HK-2 cells and the expression of membrane-located NR1 and phosphorylated CaMKII and extracellular signal–regulated kinase (ERK). MK801 and KN93 reduced CaMKII and ERK phosphorylation levels, while MK801, but not KN93, reduced the membrane NR1 signal. The levels of phosphorylated CaMKII and ERK also increased in kidneys with obstruction but were decreased by NR1 knockdown. The 4-week administration of DXM preserved renal cortex volume in kidneys with moderate ischemic–reperfusion injury.

**Conclusions:**

NMDAR participates in both acute and chronic renal fibrogenesis potentially via CaMKII-induced ERK activation.

## Background

Acute kidney injury (AKI) affects approximately 20% of hospitalized patients [1]. A proportion of patients with AKI undergo the maladaptive repair of their kidneys. This contributes to the ongoing fibrotic processes that progress over time to chronic nephropathy. Chronic kidney diseases (CKDs) have a prevalence of 10.8% in China [2] and 14.0% in the USA [3]. Renal fibrosis (RF) is a common outcome of progressive AKI nephropathy for nearly all types of CKDs [4, 5]. Clinical studies have demonstrated that renal function correlates more closely with fibrosis compared with glomerular damage [6] and is a major exacerbating factor for renal dysfunction [7]. After kidney damage, the fibrotic process is initiated with impaired kidney repair, sustained inflammation, activation of myofibroblasts, and accumulation of extracellular matrix (ECM) [5, 8]. Alpha-smooth muscle actin (α-SMA) is often used as a marker of myofibroblast formation [9]. Type I collagen (COL1A1) and fibronectin indicate enhanced deposition of ECM during fibrogenesis [10, 11]. S100 calcium–binding protein A4 (S100A4), also called fibroblast-specific protein 1, is considered a specific marker of fibroblasts in tissue remodeling [12]. The transition of tubular epithelial cells to cells with mesenchymal features, also known as epithelial-to-mesenchymal transition (EMT), is observed during fibrosis [13]. Increased expression of transcription factors associated with EMT correlates with disease progression [14, 15]. Snail is a prominent inducer of EMT and E-cadherin loss is one of the hallmarks of EMT [16]. The pathological changes that occur during RF eventually lead to renal failure [17]. Hence, therapeutically targeting RF may be a promising strategy to treat kidney diseases. At present, no effective treatment strategies are currently available.

*N*-methyl-D-aspartate receptor (NMDAR) is an ionotropic glutamate receptor. It has been well studied in the central nervous system (CNS) and has a vital role in development, learning, and memory. Besides, it can induce Ca^2+^ overload during multiple pathological conditions [18, 19]. Functional NMDAR is a tetrameric complex consisting of two NR1 subunits and two NR2 and/or NR3 subunits. The subunits are encoded by seven genes: one for NR1, four for NR2 (A–D), and two For NR3 (A–B) [20]. All subunits have a conserved domain organization, including an extracellular amino-terminal domain, an extracellular ligand–binding domain, a transmembrane domain, and an intracellular carboxy–terminal domain [20, 21]. The obligatory NR1 subunit binds glycine and D-serine and is found to have high expression in the kidneys, bone, heart, and other tissues, besides the CNS. Hence, the functional role of NMDAR outside the CNS has garnered research interest [21]. Growing evidence suggests that NMDAR is vital in numerous processes such as proliferation, apoptosis, cell adhesion and migration, actin rearrangement, cell growth and differentiation, and regulation of hormone secretion. In the kidney, the expression of NMDAR has been detected in glomeruli and tubules [22, 23]. The expression of NMDAR is induced by various kidney pathological processes, including acute ischemia–reperfusion injury (IRI) [24, 25] and diabetic nephropathy [26–28]. Whether the expression of NMDAR is associated with RF is yet to be deciphered [29].

The expression of NR1 was higher in kidney fibrotic biopsy samples than in kidneys from healthy donors (unpublished data). Hence, this study was performed to understand the mechanistic role of NMDAR in acute fibrogenesis using models of in vivo ureter obstruction and in vitro TGF-β administration. Furthermore, the mice were administered with dextromethorphan hydrobromide (DXM), which is widely used in the clinic as an NMDAR inhibitor, to understand the role of NMDAR in vivo. Then, the effect of DXM on chronic fibrosis after IRI was observed.

## Methods

### Animals

Eight-week-old C57BL/6 mice (weighing 20–25 g, 50% male) were purchased from the Experimental Animal Center in Zhejiang Medical Academy of Sciences and housed in a temperature-controlled room with 12-h day/night cycles. The mice had free access to standard food and water throughout the study. All animal studies were done in compliance with the regulations and guidelines of Zhejiang University institutional animal care and conducted according to the AAALAC and the IACUC guidelines (Permit Number: 2016–205, date of approval: 26 February 2016). Totally 60 mice were included. The mice were euthanized with an overdose of pentobarbital sodium (500 mg/kg, Merck, Shanghai, China) and CO_2_ incubation after experimentation.

### Retrograde ureteral lentivirus delivery and unilateral ureteral obstruction

The mice were anesthetized using 50 mg/kg pentobarbital sodium by intraperitoneal injection. They were then infused with lentivirus 7 days prior to unilateral ureteral obstruction (UUO) as previously described. Briefly, the mice were anesthetized, a midline abdominal incision was made on the left kidney, and the terminal ureters were obstructed. Then, 5 × 10^7^ IU/100 μL filter-purified scrambled shRNA (Scr-sh group, *n* = 6) or NR1-shRNA (NR1-sh Group, *n* = 6) lentivirus cocktail (forward: 5′-CACCGGTACCCATGTCATCCCAAATCGAAATTTGGGATGACATGGGTACC-3′ and reverse: 5′-AAAAGGTACCCATGTCATCCCAAATTTCGATTTGGGATGACATGGGTACC-3′) purchased from NovoBio Biotechnology Co., Shanghai, China [22] was infused through the ureters for 5 min via an intrathecal catheter attached to a micro-syringe (Hamilton, MA, USA) pump (WPI). After 7 days, the mice were re-anesthetized, and a midline abdominal incision was performed on the left kidney. The left ureters were double ligated with 4–0 silk surgical sutures [30].

### Human proximal tubule (HK-2) cell culture and drug treatment

HK-2 cells (American Type Culture Collection, VA, USA) were grown in keratinocyte-serum free medium (Thermofisher, MA, USA) with 10% fetal bovine serum in a 5% CO_2_ humidified incubator at 37 °C. The cells were then treated with recombinant human TGF-β1 (2 ng/mL, R&D System, MN, USA) for 48 h with a combination of NMDA (50 μM, Tocris, MN, USA), MK-801 (10 μM, Tocris), or KN-93 (10 μM, Tocris).

### Ischemia–reperfusion (IR) mouse model and DXM administration

The mice were anesthetized, and then a midline abdominal incision was performed on the right kidney. The right renal artery was isolated from the renal vein carefully and then clapped for 60 min. The mice were then divided randomly into the IR group (*n* = 6), low-dose dextromethorphan hydrobromide (DXM, Merck, Shanghai, China)-treated group (LD group, 1 mg/mL in drinking water, *n* = 6), moderate-dose DXM-treated group (MD group, 2 mg/mL in drinking water, *n* = 6), and high-dose DXM-treated group (HD group, 3 mg/mL in drinking water, *n* = 6). The mice were then sacrificed 28 days after IR.

### Masson’s trichrome staining, immunohistochemical, and immunofluorescent assays

Seven days after UUO, the mice were anesthetized, and the obstructed kidneys were harvested. Paraffin sections were stained with Masson’s trichrome or labeled with antibodies against TGF-β1 (Cell Signaling Technology, MA, USA), alpha-smooth muscle actin (Abcam, MA, USA), COL1A1 (Abcam), S100A4 (Abcam), or fibronectin (Abcam). Frozen sections and fixed cells were labeled with antibodies against NR1 (ThermoFisher), p- or total CaMKII (Abcam), p- or total extracellular signal–regulated kinase (ERK, Abcam), Snail (Abcam), or E-cadherin (Abcam) and then incubated with relevant secondary antibodies for immunohistochemical and immunofluorescence (Abcam) assays. The nuclei were stained with 4,6-diamidino-2-phenylindole (DAPI, ThermoFisher) for immunofluorescent assays.

### Western blotting analysis

The kidneys and cells were homogenized in RIPA lysis buffer with protease and phosphatase inhibitor cocktail (Cell Signaling Technology). Total protein was then separated by SDS-PAGE and blotted with antibodies against α-SMA, COL1A1, S100A4, fibronectin, NR1, p- or total CaMKII, and p- or total ERK (all from Abcam). The membranes were scanned and analyzed using the Gel Doc XR imaging system (Bio-Rad Laboratories, CA, USA).

### Statistical analysis

Data values were presented as mean ± standard error of the mean. The area of fibrosis and positive staining in tissue sections was measured with ImageJ software and shown as percentages. The standard analysis of variance with the Bonferroni test was performed using GraphPad Prism 6.0. The two-tailed Student *t* test was used for other data types. Differences were considered statistically significant at *P* < 0.05.

## Results

### Expression of NR1 was upregulated by ureteral obstruction

**Fig. 1:**
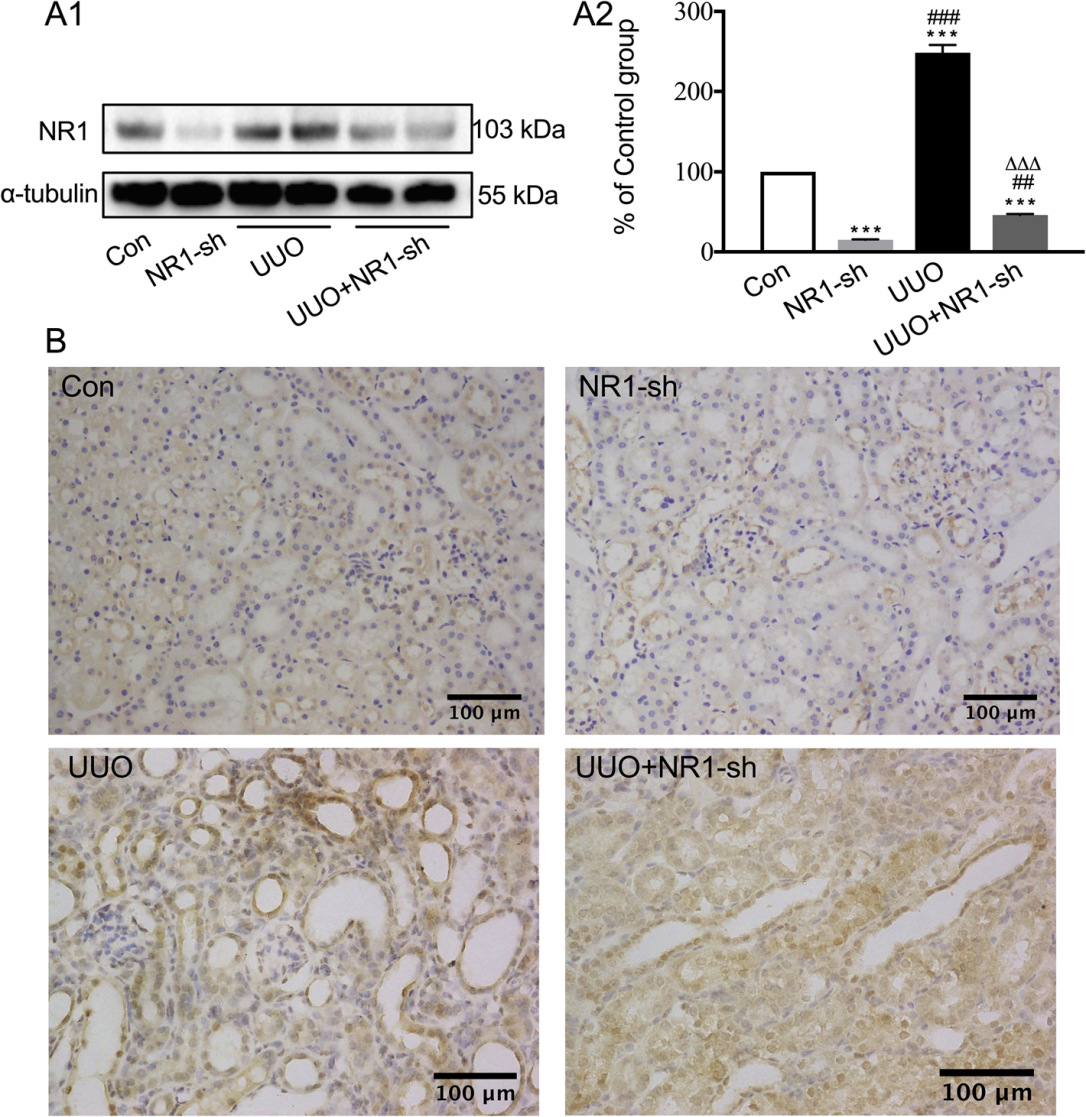
NR1 was overexpressed in UUO-injured kidneys in C57BL/6 mice. **a** Western blots of the expression of NR1 in the control (Con) group, NR1-targeting shRNA-transfected (NR1-sh) group, UUO group, and UUO + NR1-sh group (A1) and band analysis (A2). **b** Immunohistochemical staining of the expression of NR1 in the Con group, UUO group, UUO + NR1-sh group, and UUO + Scr-sh group (*n* = 6). ^***^P < 0.001 versus the Con group; ^##^*P* < 0.01, ^###^*P* < 0.001 versus the NR1-sh group; ^△△△^*P* < 0.001 versus the UUO group. Representative images from each group (original magnification: × 200; scale bar = 100 μm)

The expression of NR1 was significantly upregulated in obstructed kidneys as determined by Western blot analysis (Fig. 1a) and immunohistochemical staining (Fig. 1b). Retrograde ureteral delivery of NR1-shRNA (NR1-sh) reduced the expression of NR1 in both normal kidneys (NR1-sh vs control, *P* < 0.001). Obstruction injury increased the expression of NR1 slightly in NR1-sh-treated kidneys (UUO + NR1-sh vs NR1-sh, *P* < 0.01), but it was still much less than that in the untreated obstructed kidney (UUO + NR1-sh vs UUO, *P* < 0.001).

### NR1 knockdown suppressed the fibrotic process in obstructed kidneys

**Fig. 2:**
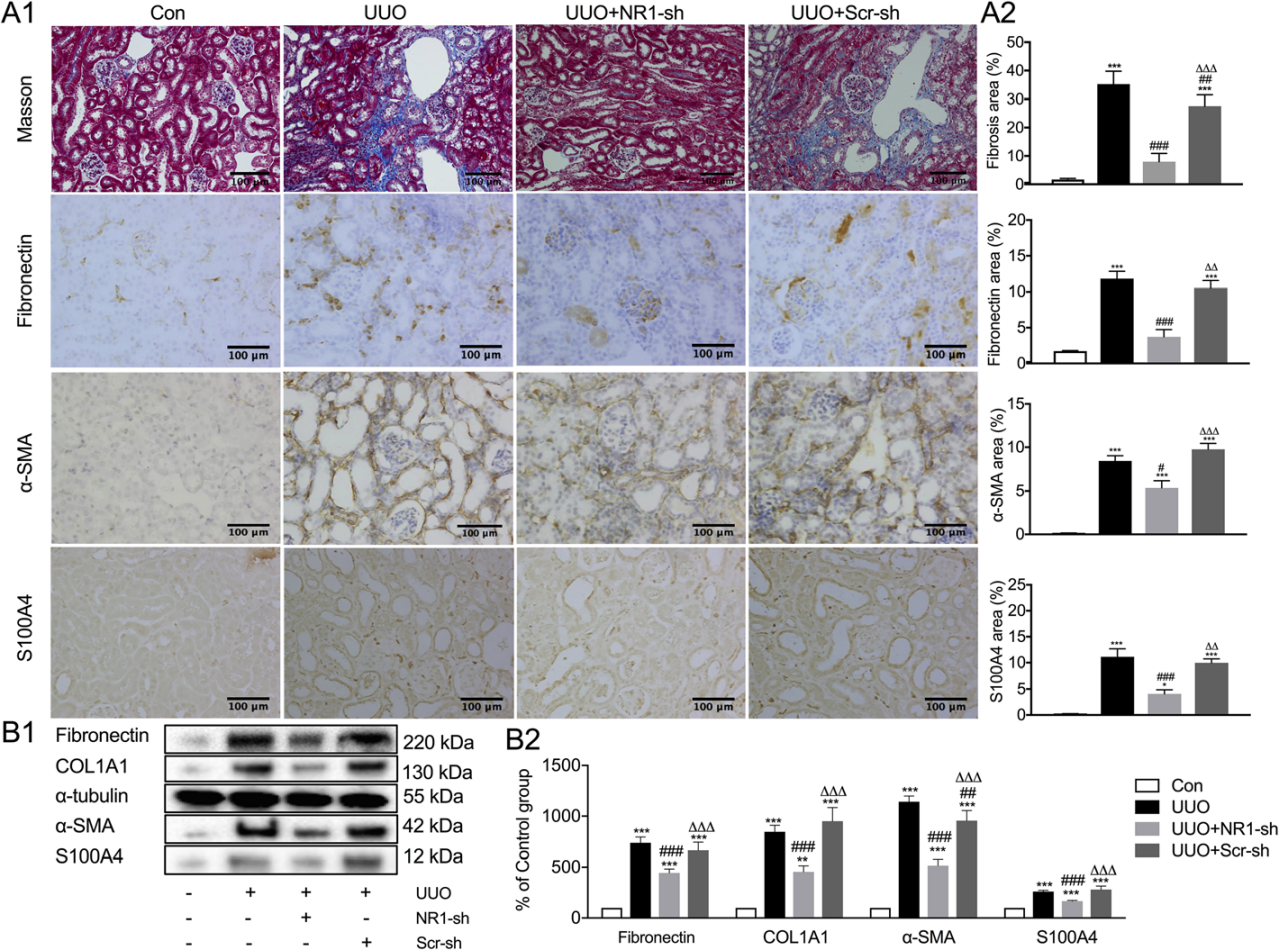
NR1 knockdown partially reversed the increased expression levels of fibrotic markers in UUO kidneys. **a** Masson’s trichrome staining of kidneys, immunohistochemical staining of fibronectin, α-SMA, and S100A4 (A1), and quantitative analysis (A2) from the control (Con) group, UUO group, UUO + NR1-targeting shRNA-transfected (NR1-sh) group, and UUO + scrambled shRNA transfected (Scr-sh) group. **b** Western blot analysis of fibronectin, COL1A1, α-SMA, and S100A4 COL1A1 (B1) and quantitative analysis (B2) (*n* = 6). ^*^P < 0.05, ^**^P < 0.01, ^***^P < 0.001 versus the Con group; ^#^*P* < 0.05, ^##^*P* < 0.01, ^###^*P* < 0.001 versus the UUO group; ^△△^*P* < 0.01, ^△△△^*P* < 0.001 versus the UUO + NR1-sh group. Representative images from each group (original magnification: × 200; scale bar = 100 μm)

The increased interstitial fibrosis in the obstructed kidneys was determined using Masson’s trichrome staining, and the upregulation of the expression of α-SMA, S100A4, fibronectin, and COL1A1 was measured with immunohistochemical staining and Western blot analysis (Fig. 2). NR1 knockdown reduced the number of collagen fibers increased by obstruction significantly compared with both UUO and Scr-sh groups (UUO + NR1-sh vs UUO, *P* < 0.001; UUO + NR1-sh vs UUO + Scr-sh, *P* < 0.01). The expression of α-SMA, S100A4, fibronectin, and COL1A1 in the obstructed kidney was also significantly reduced by NR1 knockdown.

### NR1 knockdown reduced EMT changes after obstruction

**Fig. 3:**
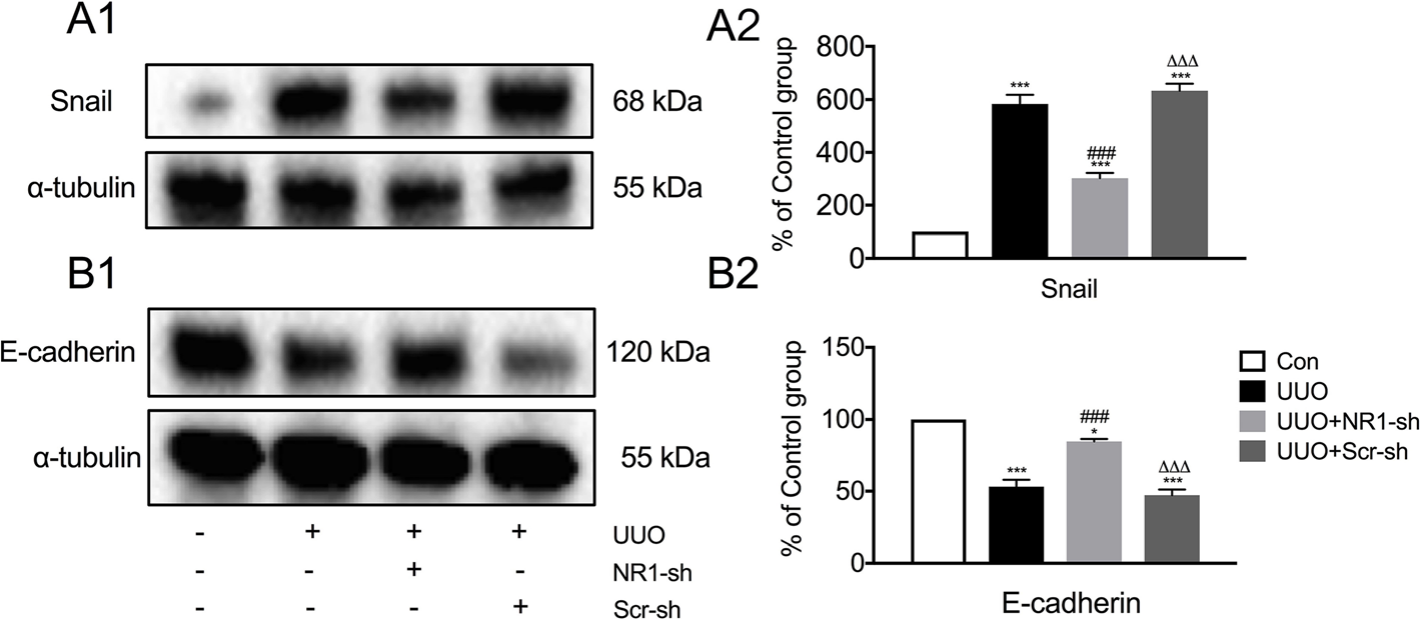
NR1 partially inhibited the changes in the expression of EMT markers after UUO injury. **a** Expression of Snail changes measured by Western blot analysis in the control (Con) group, UUO group, NR1-targeting shRNA-transfected (NR1-sh) + UUO group, and scrambled shRNA transfected (Scr-sh) + UUO group (A1) and quantitative analysis (A2). **b** Expression of E-cadherin (B1) and quantitative analysis (B2); *n* = 6; ^*^P < 0.05, ^***^P < 0.001 versus the Con group; ^###^*P* < 0.001 versus the UUO group; ^△△△^*P* < 0.001 versus the UUO + NR1-sh group

The expression of Snail increased while the expression of E-cadherin decreased sharply in obstructed kidneys in the UUO and Scr-sh groups (all *P* < 0.001); no significant differences were found between the two groups (*P* > 0.05, Fig. 3). With NR1-shRNA treatment, limited fluctuations in the expression of Snail and E-cadherin were observed in the obstructed kidney, but the expression still changed significantly compared with that in the control group (*P* < 0.001 and *P* < 0.05). The expression levels of Snail in the NR1-sh group were still higher compared with those in the normal kidneys, while the expression levels of E-cadherin were lower.

### NMDAR inhibition reduced ERK phosphorylation through CaMKII in HK-2 cells

**Fig. 4:**
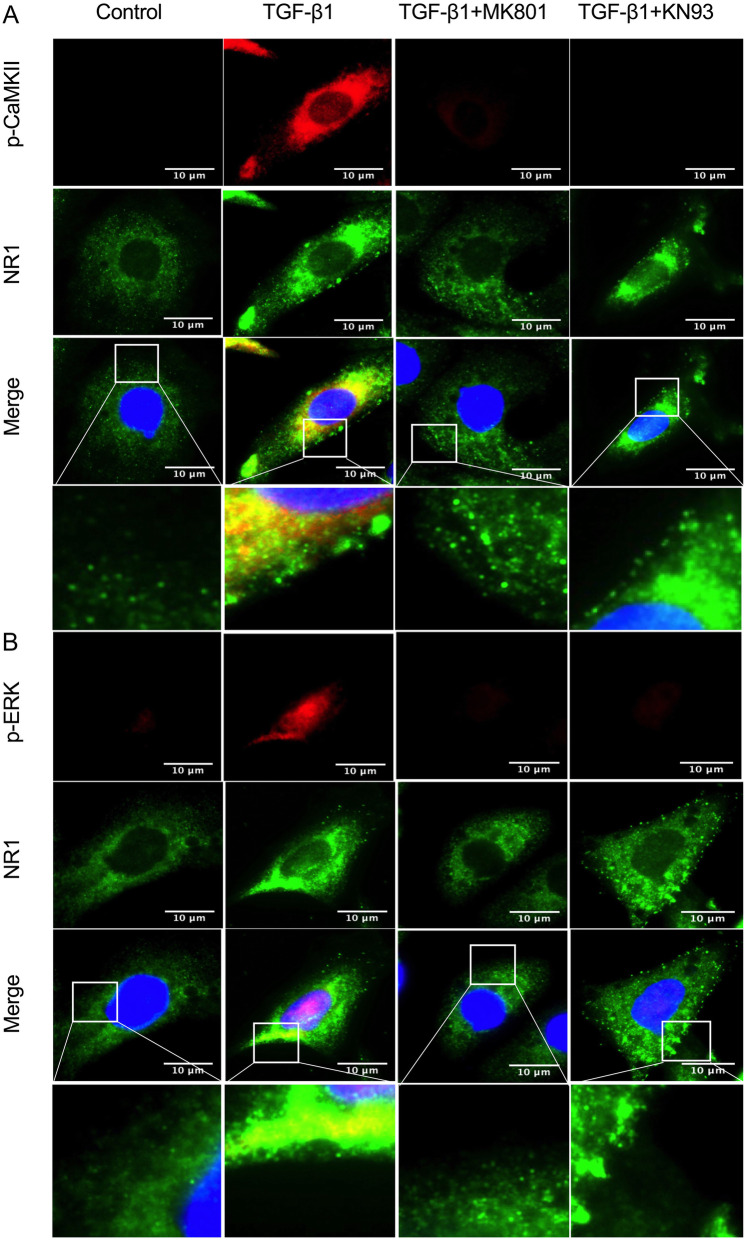
Immunofluorescence images for (**a**) the expression of p-CaMKII (red) and NR1 (green) and (**b**) the expression of p-ERK (red) and NR1 (green) in HK-2 cells in the normal control, TGF-β, TGF-β + MK801, and TGF-β + KN93 group (scale bar = 10 μm)

The fluorescence staining of p-CaMKII and p-ERK was performed in cultured tubular cells (Fig. 4). After incubation with TGF-β1 for 48 h, the elongation phenotype was observed in HK-2 cells, and the expression levels of membrane-located and cytosolic NR1, p-CaMKII, and p-ERK significantly increased. NMDAR inhibition by MK-801 reduced the expression level of membrane-located NR1 slightly and also decreased the expression levels of p-CaMKII and p-ERK significantly. However, the cytosolic region NR1 signal still increased compared with that in control cells. CaMKII inhibition by KN93 decreased the expression levels of p-CaMKII and p-ERK, but had no effect on the expression of both membrane-located and cytosolic NR1 compared with that in the TGF-β1-treated cells.

### NR1 knockdown inhibited CaMKII/ERK activation in obstructed kidneys

**Fig. 5:**
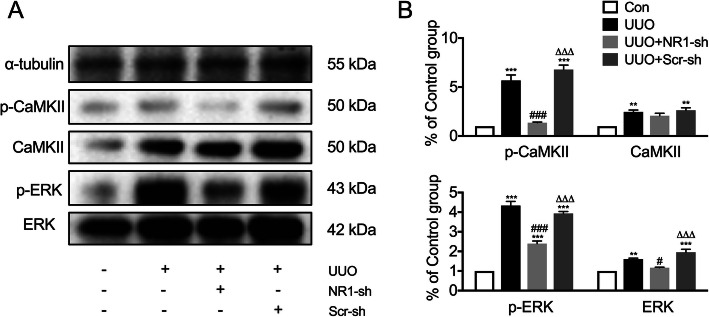
NMDAR activated CaMKII/ERK in C57BL/6 mice with UUO-induced renal fibrosis. **a** Western blots for the expression levels of total and p-CaMKII and total and p-ERK and (**b**) band analysis; *n* = 6; ^**^P < 0.01, ^***^P < 0.001 versus the Con group; ^###^*P* < 0.001 versus the UUO group; ^△△△^*P* < 0.001 versus the UUO + NR1-sh group

CaMKII and ERK and their phosphorylation levels were measured by Western blot analysis (Fig. 5). Total expression levels of CaMKII and ERK were higher in the obstructed kidneys compared with the control kidneys (*P* < 0.01). The levels of p-CaMKII and p-ERK were also significantly higher in the UUO and Scr-sh groups compared with the control groups (*P* < 0.001), but with no significant differences between the two groups (*P* > 0.05). NR1-shRNA administration significantly reduced the increase in the expression levels of both p-CaMKII and p-ERK (*P* < 0.001), but the expression level was still slightly higher than that in the control group.

### Dextromethorphan hydrobromide suppressed the chronic fibrosis process after IR injury

**Fig. 6:**
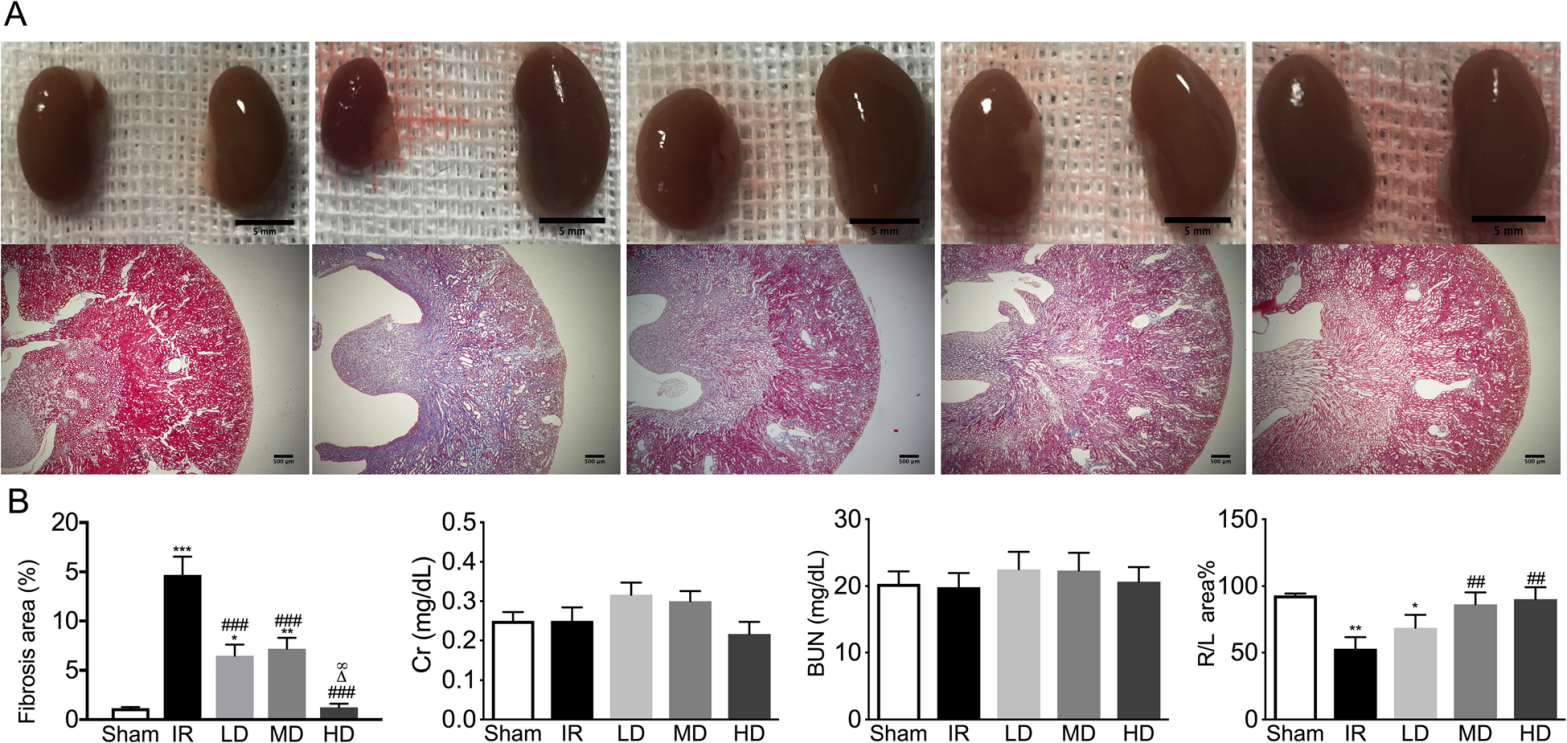
Oral NMDAR inhibitor dextromethorphan protected kidneys from chronic RF after IR injury. **a** Injured kidneys on the left and healthy kidneys on the right, with Masson’s staining of injured kidneys. **b** Quantitative analysis of fibrosis, serum creatinine (SCr), blood urea nitrogen (BUN), and volume ratios of right/left kidneys. IR group, ischemia–reperfusion group; LD group, low-dose DXM-treated group; MD group, moderate-dose DXM-treated group; HD group, high-dose DXM-treated group. Representative image of *n* = 6 individual samples per group. The original magnification of images was 40× (scale bar = 100 μm)

Moderate ischemic–reperfusion injury was made to induce the chronic RF process. Different dosages of DXM, the NMDAR inhibitor, were given in the drinking water for 4 weeks after reperfusion injury. As shown in Fig. 6, the suppressed fibrosis process was observed in the treated groups compared with untreated IR group. The volumes of ischemic kidneys significantly reduced, the persevered cortex area sharply decreased, and the fibrotic area remarkably increased in the IR group (*P* < 0.001 vs sham). In the low (LD) and medium (MD) dose groups, the volumes of ischemic kidneys were still smaller than those of contralateral kidneys, but significantly larger than those of the untreated ischemic kidneys in the IR group. In the high (HD) dose group, the volumes of ischemic kidneys were similar to those of contralateral kidneys, and had no significant difference compared with those of the ipsilateral kidneys in the normal (sham) group. The serum creatinine (SCr) and blood urea nitrogen (BUN) levels had no difference between the four groups (*P* > 0.05).

## Discussion

The findings of the present study were as follows. (1) NMDAR was overexpressed during RF induced by ureteral obstruction in vivo and by TGF-β treatment in vitro. (2) NMDAR activation induced the phosphorylation of CaMKII and ERK. (3) Both NR1 inhibition and knockdown significantly reduced the phosphorylation levels of CaMKII and ERK. (4) CaMKII inhibition reduced the phosphorylation of ERK but had no effect on the expression of NR1. (5) The oral administration of NMDAR inhibitor suppressed chronic RF after sublethal ischemic injury.

Fibrosis is associated with a reduction in the functional structures of the kidney, eventually leading to organ failure. RF is the common outcome of all progressive nephropathies. The initial therapeutic strategy for the treatment of renal fibrogenesis was to target the renin–angiotensin system using angiotensin-converting enzyme inhibitors or angiotensin receptor blockers (ARB) [31]. However, this approach was not efficacious in treating RF. Later, several studies demonstrated a central role of TGF-β in fibrosis [32]. However, targeting TGF-β is problematic due to its diverse roles in cell proliferation and differentiation, wound healing, and the immune system [33]. Although several targeting strategies have been proven to be effective in RF animal models, no novel therapeutic targets have demonstrated safety and efficacy in preventing or alleviating RF in humans [34]. One important reason is that rodent models often do not fully mimic the human clinical situation and only a few studies have used more than one model to verify their findings [35]. Multiple mouse models of RF based on different inducements are available. Surgical animal models have been used to reduce kidney mass, UUO, and IRI. Kidney mass reduction was achieved by 5/6 nephrectomy; however, it was less likely to mimic the human clinical situation [36]. Hence, UUO and IRI are the closest models to replicate human diseases. Therefore, both these models were used in the present study to determine the efficacy of NMDAR in RF. The UUO model is the most widely used because of the rapid development of tubular atrophy, interstitial fibrosis, and matrix deposition. However, this absolute obstruction is rarely observed in humans. Nonetheless, the UUO model reproduces a typical fibrotic sequence of events, including hemodynamic changes, interstitial inflammatory infiltration, and tubular cell death [37]. The results showed that the levels of TGF-β (Supplemental Fig. 1) and NR1 increased after UUO injury, and NR1 knockdown protected kidneys from acute injury after UUO with stable expression of TGF-β (Supplemental Fig. 1). A 60-min ischemia induces severe hypoxia and cell damage [38], and is exacerbated by reperfusion, which eventually leads to fibrosis [39]. Hence, the IRI model was used to mimic chronic progression from injury to fibrosis. Ischemic kidneys from DXM-treated mice had reduced histological features 28 days after reperfusion. The effect of DXM on renal fibrosis was dose dependent, with kidneys from mice treated with a high dose of DXM exhibiting no significant differences compared with kidneys from the control mice.

EMT of tubular epithelial cells (TECs) is a feature observed during renal fibrosis [13]. TEC injury results in the loss of functional parenchyma and induces pathological processes including EMT [13]. Injury-induced EMT eventually leads to fibrosis [40]. Preventing the initiation of EMT results in the reduction of myofibroblast recruitment and extracellular matrix deposition, and hence preserves functional TECs and improves organ function [13]. This study demonstrated that the levels of EMT markers were altered after UUO-induced injury along with NMDAR activation. NR1 knockdown reduced the changes in the expression levels of Snail and E-cadherin, suggesting that NMDAR was a target for EMT inhibition.

In addition, the present study investigated the downstream pathway of NMDAR using HK-2 cells treated with TGF-β treatment as an in vitro model (Supplemental Fig. 2). Immunofluorescent assays demonstrated low expression levels of NR1 in the cytoplasm and even lower levels on the membranes of untreated HK-2 cells. The cytoplasmic localization of NR1 is associated with its production in the endoplasmic reticulum and maturation, while membrane anchoring is associated with the maturation and function of NMDARs [41]. TGF-β increases the expression and localization of NR1 both in the cytoplasm and on the membrane. CaMKII is one of the key protein kinases mediating changes in intracellular Ca^2+^ levels [42]. CaMKII phosphorylation increases significantly during fibrogenesis and is crucial in TGF-β-induced fibrogenic cascades [43]. Besides fibrosis, CaMKII mediates oxidative stress, which is pivotal for IRI progression [44]. CaMKII has been demonstrated to be activated by NMDAR in the CNS as an intracellular sensitive kinase [45]; however, its function in the kidney has not been deciphered. The present study demonstrated that CaMKII was phosphorylated by activated NMDAR and NR1 inhibition reduced CaMKII phosphorylation. In the UUO model, NR1 knockdown reduced CaMKII phosphorylation but had no effect on the total expression of CaMKII.

ERK is a widely expressed intracellular signaling protein kinase in diverse biological functions [46]. The phosphorylation at Thr^202^/Tyr^204^ residues results in ERK activation. ERK has been demonstrated to be involved in RF, but its role has been controversial. A majority of studies have shown that ERK acts as a pro-fibrotic factor important for inflammatory responses [47], TGF-β/Smad signaling [48], and ECM and myofibroblast accumulation [49, 50]. In addition, ERK has recently been shown to participate in EMT progression, and the inhibition of ERK ameliorates renal interstitial fibrosis by suppressing tubular EMT [51, 52]. However, Jang et al. demonstrated that the activation of ERK accelerated renal tubular epithelial cell repair and inhibited fibrogenesis following IRI [53]. This result was consistent with previous findings that the phosphorylation of ERK was protective and promoted the growth of renal tubular epithelium but induced apoptosis in renal fibroblasts [54]. ERK was found to be activated on CaMKII phosphorylation after the overexpression of NRI using both in vitro and in vivo fibrosis models. NR1 shRNA knockdown or inhibition with MK801 reduced the phosphorylation levels of ERK. The inhibition of CaMKII reduced ERK phosphorylation, regardless of the expression of NR1 in the cytoplasm and on the membrane.

## Conclusions

In summary, NMDAR participates in renal fibrogenesis by activating the CaMKII/ERK pathway. NMDAR inhibition via oral administration is promising in protecting against fibrosis after IRI. It is presumed that NMDAR is a potential therapeutic target. However, more studies are required to substantiate the findings.

## Supplementary information

**Additional file 1: Supplemental Fig. 1.** Immunohistochemical staining of TGF-β1 in kidneys from the control (Con) group, UUO group, UUO + NR1-sh group, and UUO + Scr-sh group (A) and quantitative analysis (B). Representative image of *n* = 6 individual samples per group. ^*^P < 0.05, ^**^P < 0.01, ^***^P < 0.001 versus the sham group; ^##^*P* < 0.01, ^###^*P* < 0.001 versus the IR group; ^△^*P* < 0.05 versus the LD group; ^∞^*P* < 0.05 versus the MD group. Original magnification: × 200. Scale bar = 100 μm.

**Additional file 2: Supplemental Fig. 2.** Immunofluorescence images for the expression of α-SMA in HK-2 cells in the normal control, TGF-β1, TGF-β1 + MK801, and TGF-β1 + KN93 groups (scale bar = 10 μm).

## Data Availability

The datasets used and/or analyzed in the present study are available from the corresponding author on reasonable request.

## Abbreviations

AKI: Acute kidney injury
CaMKII: Ca^2+^/calmodulin-dependent protein kinase II
CKD: Chronic kidney disease
DXM: Dextromethorphan hydrobromide
ECM: Extracellular matrix
EMT: Epithelial-to-mesenchymal transition
IR: Ischemia–reperfusion
NMDARs: *N*-methyl-D-aspartate receptors
α-SMA: α-Smooth muscle actin
UUO: Unilateral ureteral obstruction
