# Supplementary figures and images for: NMDA receptor-mediated CaMKII/ERK activation contributes to renal fibrosis
Source: BMC Nephrol. 2020 Sep 9;21:392. doi: 10.1186/s12882-020-02050-x (PMC7488001; doi:10.1186/s12882-020-02050-x)

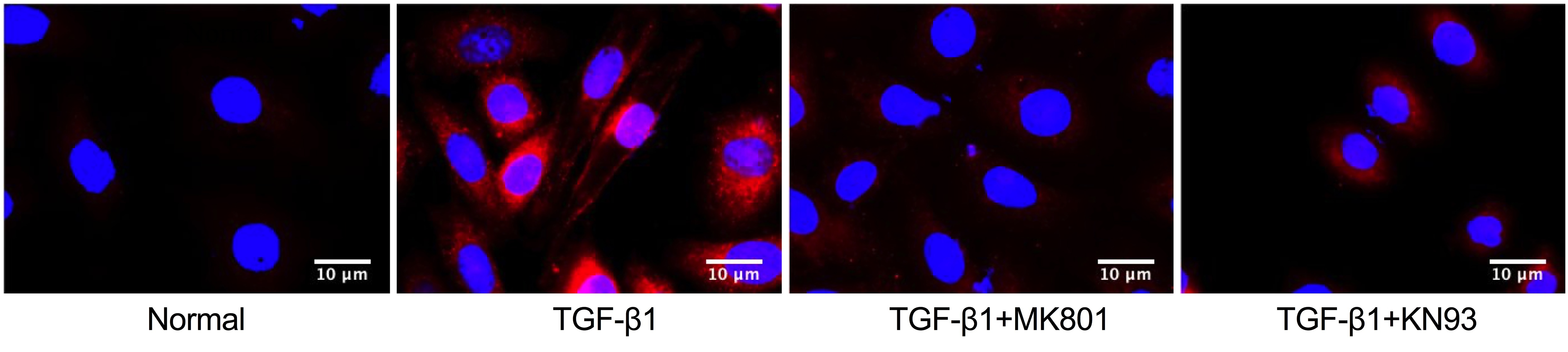

Supplement: Supplementary file 2 — Additional file 2: Supplemental Fig. 2. Immunofluorescence images for the expression of α-SMA in HK-2 cells in the normal control, TGF-β1, TGF-β1 + MK801, and TGF-β1 + KN93 groups (scale bar = 10 μm). [file 12882_2020_2050_MOESM2_ESM.jpg]
